# Whole-Exome Sequencing for the Identification of Genetic Factors Implicated in Severe Bacterial Infections: A Systematic Review

**DOI:** 10.1093/infdis/jiag006

**Published:** 2026-01-07

**Authors:** Morgane Gélin, Élise Launay, Nicolas Vince

**Affiliations:** Nantes Université, INSERM, Ecole Centrale Nantes, CHU Nantes, Center for Research in Transplantation and Translational Immunology, UMR 1064, Nantes, France; Department of Pediatrics and Pediatric Emergency, Hôpital Femme Enfant Adolescent, CHU de Nantes, Nantes, France; Nantes Université, INSERM, Ecole Centrale Nantes, CHU Nantes, Center for Research in Transplantation and Translational Immunology, UMR 1064, Nantes, France; Department of Pediatrics and Pediatric Emergency, Hôpital Femme Enfant Adolescent, CHU de Nantes, Nantes, France; Center of Clinical Research Femme Enfant Adolescent, Inserm 1413, Nantes University Hospital, Nantes, France; Inserm UMR 1153, Obstetrical, Perinatal, and Pediatric Epidemiology Research Team (Epopé), Center of Research in Epidemiology and Statistics, Paris University, Paris, France; Nantes Université, INSERM, Ecole Centrale Nantes, CHU Nantes, Center for Research in Transplantation and Translational Immunology, UMR 1064, Nantes, France

**Keywords:** Whole-exome sequencing, severe bacterial infections, inborn errors of immunity, previously healthy patients, systematic review

## Abstract

**Background:**

Severe bacterial infections (SBIs) represent a major health issue worldwide. Many studies have explored patients’ genetic predispositions to SBI, but most of them chose a candidate-gene design. Only few adopted a whole-exome sequencing (WES) approach. We aimed at reporting nontargeted WES studies describing genetic variants associated with SBI susceptibility in previously healthy patients without a known predisposition for infections.

**Methods:**

We included studies using WES in previously healthy patients who had SBI. We excluded studies that included nonbacterial infections or patients with a known genetic or dysimmune disorders. We assessed certainty in the body of evidence and detected risk of bias. Studies were grouped according to the patients' infectious phenotype to present main common characteristics and compare results.

**Results:**

Twelve studies were included, gathering 694 patients with WES data. They described genetic associations with various infectious phenotypes, using heterogenous methods to prioritize genetic variants. This diversity led to the identification of different genes or pathways associated with infection susceptibility or severity, supporting WES use in patients with SBI. WES was also a performant diagnostic tool. In this review, 42% of previously healthy patients with SBI had putatively disease-causing variants in genes with inborn errors of immunity.

**Conclusions:**

Overall, included studies supported the use of WES as they successfully diagnosed inborn errors of immunity in patients with SBI. Future studies should follow strict guidelines to correctly prioritize disease-causing variants. Because of the rarity of this disease, sample sizes are often limited. Collaboration between research teams should allow for large-scale studies with robust statistical results.

## Teaser

We gathered 12 well-conducted studies analyzing genetic predisposition to various severe bacterial infection (SBI) phenotypes. Next-generation sequencing in this context identifies new associations with genes not yet linked to SBI and the diagnostic performance of whole-exome sequencing.

## Graphical Abstract

**Graphical Abstract:**
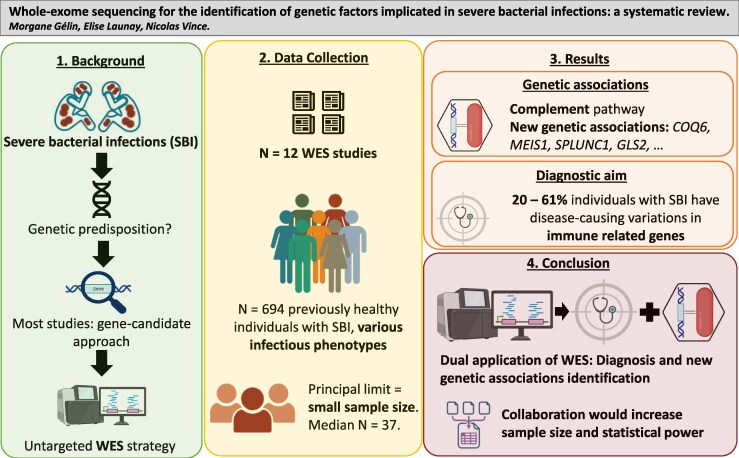

Severe bacterial infections (SBIs) represent a major public health issue, and, despite progress in diagnosis, management, and vaccination, they still have high mortality and morbidity rates worldwide. In 2017, it was estimated that infection-related sepsis represented 33 million cases worldwide and was responsible for >6 million deaths [1]. Sepsis-related mortality rates peak in childhood and older ages, with an estimated 2.9 million deaths worldwide in 2017 among children <5 years of age [1]. Understanding the underlying causes of SBI is an essential element to limit occurrences and consequences of these diseases, allow the identification of novel therapeutic targets, and improve prevention and clinical management to decrease mortality and morbidity rates.

All patients are not equal when facing bacterial infection. Some may have anatomic conditions increasing the risk of severe infection (meningeal breach or anatomic asplenia) or acquired immunodepression due to human immunodeficiency virus or medical conditions requiring immunosuppressive treatments. Inborn errors of immunity (IEI) are known predispositions to the development of severe infections [2], due to many different possible impairments in the immune system leading to an inappropriate response to pathogen aggression. It has become clear that genetic factors could also be involved in susceptibility to sepsis or SBI [3].

Many studies have explored these potential genetic susceptibilities and have led to the discovery of numerous genetic defects influencing a host's ability to fight against a bacterial infection [4]. To better understand these essential findings, published systemic reviews have collected studies about SBI or sepsis genetic susceptibility, with a focus on specific pathogens such as meningococcus [5, 6] or pneumococcus [7]. As stated by Kloek et al [7] and Hodeib et al [6], the vast majority of these previous studies used a candidate gene approach; that is, they chose to investigate only 1 gene or a few already known to be potentially linked to disease development [6, 7]. This approach, while very effective at discovering novel variants in these genes, cannot permit the identification of variants in undocumented genes, by definition not yet identified as linked to SBI.

Here, in this systematic review, we aimed to report nontargeted whole-exome sequencing (WES) studies describing genetic variants associated with SBI susceptibility in previously healthy patients without a known predisposition for infections. The secondary objectives were to assess the association between these variants and disease severity and to analyze the methods used. Indeed, the increasing availability of next-generation sequencing (NGS) technologies has enhanced untargeted approaches for this field of research [8]. WES and whole-genome sequencing, untargeted approaches, aim to expand the quest for disease-causing variations to coding regions or the entire genome, respectively. Only a few studies selected these untargeted approaches, and they were usually applied to patients with known IEI without genetic diagnoses, such as common variable immune deficiency or severe combined immune deficiency [8].

## METHODS

We performed a systematic review of studies that used WES in patients diagnosed with SBI without prior signs of immune deficiency or conditions increasing the risk of SBI and that they explored these patients’ genetic susceptibility to disease development. We followed the PRISMA (Preferred Reporting Items for Systematic reviews and Meta-Analyses) guidelines for reporting these studies [9] (Supplementary Table 1). Search strategy, precise inclusion and exclusion criteria, and screening methods are detailed in the Supplementary materials.

We produced descriptive statistics to summarize information extracted from the studies. When applicable, we grouped studies evaluating the same infectious phenotype and described their results together. Methods were carefully read to assess the possibility of comparisons between studies. Meta-analysis was not possible because of the heterogeneity of the selected studies in terms of study design, population phenotypes, variant prioritization methods, and measured outcomes. Descriptions of individual studies are displayed in Tables 1–3. Studies were evaluated for bias, and we assessed the certainty in the body of evidence for each study. Methods used for this assessment are detailed in the Supplementary materials.

**Table 1.** Population Description for Each Study

Authors, Year [Reference]	Patient Country of Origin	Patient Phenotype	Inclusion Design	Patients, No.	Control Description	Controls, No.
Asgari et al, 2016 [10]	Switzerland and Australia	Sepsis due to community-acquired *Pseudomonas aeruginosa* in children	Prospective and retrospective	11	In-house controls	533
Taudien et al, 2016 [11]	Greece and Germany	Group B: adult patients with severe sepsis, without predisposing conditions, critically ill despite receiving appropriate therapy	Prospective in biocollection; retrospective selection for this study	27	Group A: adult patients with less severe sepsis, who survived after 28 d despite empirical administration of inappropriate antimicrobials	32
Salas et al, 2018 [12]	Spain	Pneumococcal pulmonary empyema in children	Prospective	8	IBS from 1KG project and Spanish exome data from Dopazo et al [22]	157 (IBS from 1KG project) and 267 (Dopazo et al) [22]
Scott et al, 2018 [13]	United States	Complicated *Staphylococcus aureus* bacteremia in adult patients	Prospective	84	Patients with uncomplicated *S. aureus* bacteremia	84
Borghesi et al, 2020 [14]	Switzerland	Sepsis with bacteremia (pneumococcus, *S. aureus*, group A or B streptococcus, meningococcus, *Haemophilus influenzae*) in previously healthy children	Prospective	176	In-house controls	519
Mashbat et al, 2020 [15]	United Kingdom	2 Siblings with IMD and 186 other patients with IMD for targeted sequencing	Prospective	WES in 2 siblings and targeted sequencing in 186 other patients	In-house database of children with life-threatening infections	549
Kernan et al, 2022 [16]	United States	Children with severe sepsis admitted in PICU	Prospective	401 Total; WES in 330	gnomAD population	141 456
Bendapudi et al, 2024 [17]	United States	Adult patients with purpura fulminans	Retrospective	37	Patients with sepsis with lower disease severity	87
Hassan et al, 2023 [18]	Pakistan	Recurrent typhoid fever in an adult patient	Case report	1	In-house exome database	Not indicated
Altammar et al, 2024 [19]	Kuwait	Severe recurrent bacterial infections in a child	Case report	1	NA	NA
Zhang et al, 2024 [20]	China	*P. aeruginosa* sepsis and death in a child	Case report	1	Family members	Not indicated
Walker et al, 2024 [21]	Papua New Guinea	Pneumococcal acute lower respiratory tract infection in children	Prospective	6	Family members	13

Abbreviations: 1KG, 1000 Genomes Project; gnomAD, Genome Aggregation Database; IBS, Iberian populations in Spain; IMD, invasive meningococcal disease; NA, not applicable; PICU, pediatric intensive care unit; WES, whole-exome sequencing.

**Table 2.** Variant Prioritization Methods and Study Results

Authors, Year [Reference]	Patients, No.	Validation	Replication	Family Analysis	Variant Prioritization	Gene Panels	Results	Correction for Multiple Testing
Asgari et al, 2016 [10]	11	Functional analysis for 1 patient	…	7 Trios	Rare variants: MAF <1%, LoF variants, zygosity, GSEA	PID gene panel (n = 252)	12 Potentially disease-causing variants, including 2 novel variants in known PID genes: *BTK* and *DNMT3B*; targeted search for variants in known PID genes: significant enrichment of complement pathway cluster	…
Taudien et al, 2016 [11]	27	Sanger sequencing and functional analyses	15 German patients, 5 in group A and 10 in group B	…	Rare variants: MAF <0.5%, SIFT, PolyPhen-2, Grantham scores, stop-gain/loss, splice donor/acceptor SNVs, GSEA	…	Newly developed Sem-SCM model from various identified pathways; best model: 77.8% sensitivity, 75% specificity, and 76.3% accuracy; model validated on replication population	…
Salas et al, 2018 [12]	8	Analysis of transcriptomic data	Replication using different European control groups	…	Common variants: MAF >5%, then rare variants; CADD/DANN algorithm, burden test with DANN as covariate GSEA	…	Significant association with 2 variants in *MEIS1* 5' UTR and *TSPAN15* 3’ UTR; burden test: 3 genes with significant *P* values: *OR9G9*, *MUC3A,* and *MUC6*; burden test on rare variants: 4 genes with significant *P* value: 3 previous genes and *APOB*; test of these genes as potential biomarkers by analyzing transcriptomic data from other studies (gene expression in humans and mice with pneumococcal keratitis): AUC >75% for *MEIS1*, *TSPAN15*, and *APOB*	Bonferroni correction
Scott et al, 2018 [13]	84	Functional analysis	240 Patients with *Staphylococcus aureus* bacteremia, 122 complicated and 118 uncomplicated; targeted sequencing	…	CADD, SeattleSeq, missense, nonsense, splice-gain or loss variations, burden test	…	No SNV was associated with SAB; gene-based burden test: no significant association; replication study: no SNV was significantly associated with SAB; *GLS2* gene was significantly associated with SAB in gene-based burden test (after Bonferroni correction for 342 tested genes)	Bonferroni correction
Borghesi et al, 2020 [14]	176	…	…	…	Rare variants: MAF <1% homozygous variants in gnomAD, MAF <0.01% heterozygous variants in gnomAD, MAF <1% in in-house database; CADD score	PID genes (n = 240)	41 Rare and pathogenic variants in 24 PID genes in 35 patients (20%); no significant correlation between clinical or laboratory findings of patients with or without PID variants	…
Mashbat et al, 2020 [15]	WES in 2 siblings and targeted sequencing in 186 other patients	Sanger sequencing and functional analyses	…	Single family	Rare variants: MAF <1% or absent in public databases; CADD, SIFT, gene damage index, SHAPEIT method used to obtain IBD segments	…	231 Rare, nonsynonymous variants, predicted high impact on protein, after exclusion of variants falling outside IBD blocks in 2 siblings; prioritization of *SPLUNC1* after literature reading (has a known impact on defense against gram-negative bacteria); another patient with IMD has the same variant in *SPLUNC1*; mutant *SPLUNC1* showed reduced antibiofilm activity, increased Nm adhesion, and increased invasion of cells	…
Kernan et al, 2022 [16]	401 Total; WES in 330	…	…	…	Rare variants: MAF <5%; HGMD (Qiagen), novel null variants in disease, consistent inheritance pattern (ACMG/AMP classification)	IEI genes (n = 430)	More than half of children overall and more than three-quarters of African American children had variants in IEI genes; children with variants had increased odds of positive blood or urinary culture, were more likely to be lymphopenic, hyperferritinemic, and thrombocytopenic and to have CRP >10 mg/L, and had significantly increased odds of ECMO	Benjamini-Hochberg method
Bendapudi et al, 2024 [17]	37	Functional analyses	…	…	Rare variants: MAF <5% and below an optimal in-cohort threshold; SIFT, PolyPhen-2, LoF variants; RVTT: pathway-based burden test	Complement system (n = 27), coagulation system (n = 46), and glycolytic pathway (n = 68)	No. of variants in complement pathway was independently associated with purpura fulminans, with additive effect of multiple variants to provoke purpura fulminans (*P* = .01); functional analyses on genes *CFD, CR3, CR4, ITGB2, ITGAM,* and ITGAX	…
Hassan et al, 2023 [18]	1	Sanger sequencing	…	…	Rare variants: MAF <1% in in-house exome database; SIFT, PolyPhen2, MutationTaster, CADD	IL-12/IFN-γ axis pathway (n = 25)	Identification of 2 probable disease-causing mutations in 2 genes: *IL23R* and *ZNFX1*	…
Altammar et al, 2024 [19]	1	Sanger sequencing	…	…	Rare variants: MAF <1%; frameshift, LoF variants, likely pathogenic according to ACMG/AMP classification	…	Novel *IRAK4* mutation	…
Zhang et al, 2024 [20]	1	Sanger sequencing and functional analyses	…	Yes	Variants absent in public databases, SIFT, ACMG/AMP classification	…	Two compound heterozygous variants in *IRAK4* gene	…
Walker et al, 2024 [21]	6	Sanger sequencing and functional analyses	Independent ALRI cohort (115 patients and 130 family member controls)	Yes	Rare variants: MAF <1%, CADD score, variants homozygous in ≥1 patient and no control	…	Variant rs760972463 in *COQ6* gene associated with ALRI, present in 3 of 6 patients; enriched homozygosity for this variant in replication cohort (Fisher exact test *P* = .036); validated with functional analyses	…

Abbreviations: ACMG, American College of Medical Genetics and Association for Molecular Pathology; ALRI, acute lower respiratory tract infection; AMP, Association for Molecular Pathology; AUC, area under the receiver operating characteristic curve; CADD, Combined Annotation Dependent Depletion; CRP, C-reactive protein; DANN, deleterious annotation of genetic variants using neural networks; ECMO, extracorporeal membrane oxygenation; gnomAD, Genome Aggregation Database; GSEA, gene-set enrichment analysis; HGMD, human gene mutation database; IBD, identical by descent; IEI, inborn errors of immunity; IFN, interferon; IL-12, interleukin 12; LoF, loss of function; MAF, minor allele frequency; Nm, *Neisseria meningitidis*; PID, primary immunodeficiency; RVTT, rare variant trend test; SAB, *Staphylococcus aureus* bacteremia; SIFT, sorting intolerant from tolerant; SNV, single-nucleotide variant; UTR, untranslated region; WES, whole-exome sequencing.

**Table 3.** Criteria Used to Assess Certainty in the Body of Evidence

Authors, Year [Reference]	Precise Phenotye	Family Analysis	Control Population	Similar Genetic Ancestry in Patients and Controls	Multiple Tools to Evaluate Variant Impact	Support for Genetic Implication From Published Sources	Start With Study of Known Genes	Quality Control of Genoytping	Coverage Control	Statistical Testing	Correction for Multiple Testing	Replication Analysis	Functional Analyses	Q-Genie Score
Asgari et al, 2016 [10]	x	x	x	…	…	x	x	x	x	x	…	…	x	54
Taudien et al, 2016 [11]	x	…	x	x	x	x	…	x	x	x	…	x	x	58
Salas et al, 2018 [12]	X	…	x	x	x	x	…	x	x	x	x	(x)	…	62
Scott et al, 2018 [13]	x	…	x	x	x	x	…	x	x	x	x	x	x	57
Borghesi et al, 2020 [14]	x	…	x	…	…	x	x	x	x	x	…	…	…	54
Mashbatet al, 2020 [15]	x	x	x	…	x	x	x	x	x	…	…	x	x	52
Kernan et al, 2022 [16]	X	…	x	…	x	x	x	x	x	x	x	…	…	55
Bendapudi et al, 2024 [17]	X	…	x	x	x	x	x	x	x	x	x	…	x	70
Hassan et al, 2023 [18]	CR	…	x	…	x	x	x	x	x	x	…	…	…	CR
Altammar et al, 2024 [19]	CR	…	…	…	x	x	x	x	x	…	…	…	…	CR
Zhang et al, 2024 [20]	CR	x	…	…	x	x	x	…	x	…	…	…	x	CR
Walker et al, 2024 [21]	X	x	…	x	…	…	…	x	…	x	…	x	x	52

Abbreviation: CR, case report.

This review was registered on the PROSPERO registry (identification no. 1154859). No protocol was prepared prior to its realization.

## RESULTS

**Figure 1.**
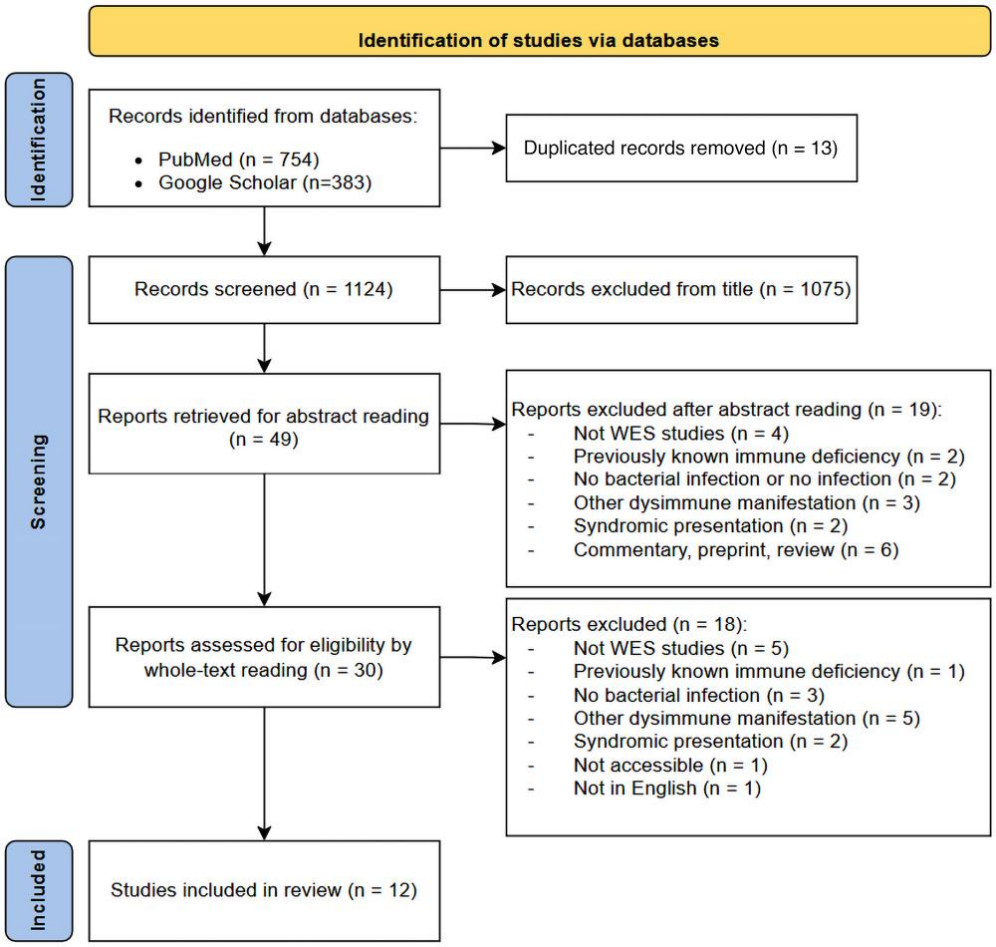
Flow diagram illustrating the selection process. Flow diagram adapted from PRISMA guidelines [9]. Abbreviation: WES, whole-exome sequencing.

After the selection process, we selected 12 studies describing WES to analyze the genetic influence on SBI development in previously healthy patients (Figure 1). They were published from 2016 to 2024. Overall, they studied genetic data from a total of 1186 SBI cases (when adding study and replication populations), including 694 patients with WES data from 11 countries. Three studies were case reports [18–20], 8 described childhood cases of severe and/or recurrent SBI [10, 12, 14–16, 19–21], and 4 studied adult patients [11, 13, 17, 18].

### Study Design

#### Population Description

Five studies focused on patients with SBI from various bacteria. Three studied severe sepsis cases [11, 14, 16], 1 reported a case of severe and recurrent bacterial infections [19], and 1 selected patients with purpura fulminans due to various bacteria [17]. Other patient populations included patients with severe and/or recurrent bacterial infection due to meningococcus (n = 1) [15], pneumococcus (n = 2) [12, 21], *Pseudomonas aeruginosa* (n = 2) [10, 20], *Staphylococcus aureus* (n = 1) [13], and typhoid fever (n = 1) [18].

Apart from case reports (n = 3), patients were included prospectively in 6 studies [12–16, 21], retrospectively in 1 [17], and following a mixed prospective and retrospective design in 2 [10, 11]. Sample sizes, replication populations set aside, varied from 2 to 401 patients (median, 37) and from 2 to 330 patients who underwent WES (median, 27).

Family analyses were performed in 4 studies [10, 15, 20, 21], including 1 case report. These analyses enabled the authors to select variants on the basis of the suspected inheritance mode of the disease (eg, autosomic recessive or dominant), depending on the affected family members.

Nine studies compared their results with those in a control population different from family members. These control populations were composed of patients with less severe infections (n = 3) [11, 13, 17], in-house exome databases (n = 4) [10, 14, 15, 18], or public databases (n = 2) [12, 16]; they varied in size from 32 to >140 000 individuals, with a median control population of 472. Population descriptions are summarized in Table 1.

#### Variant Prioritization

When using WES, many genetic variants are identified compared with a reference genome. Not all of these variants are relevant to the studied phenotype. They need to be filtered to focus on putative disease-causing ones, following various possible approaches. Various prioritization methods used in selected studies are detailed in the Supplementary materials and Table 2.

### Certainty Assessment

With high-throughput sequencing approaches such as WES, several hundred thousand genetic variations can be identified for each patient. Distinguishing pathogenic variants responsible for disease development in humans from “the broader background of variants present in all human genomes” can be challenging [23]. Reliably investigating the role of sequence variants in human disease requires high-quality studies. To that end, MacArthur et al [23] published guidelines in 2014 to investigate causality of sequence variants in human disease. We used these guidelines to assess certainty of the results presented by studies included in this review. Details of criteria used to assess certainty and confidence in the results for each study are provided in Table 3. Of note, no selected study met all selected criteria, illustrating the challenge of quality genetic analyses. Moreover, the overall methodological assessment was done using the Q-Genie scoring tool (Table 3), for all studies except case reports.

#### Populations

With respect to case reports (n = 3), most studies selected patients with a well-defined, precise homogenous phenotype and detailed inclusion criteria. In 2 studies [11, 21], case and replication populations did not have the exact same infectious phenotype, creating a risk that they would not be comparable.

Eleven studies compared variants identified in case patients and in controls. Controls could be family members (n = 2) [20, 21] or unrelated control populations (n = 9) [10–18] as we previously described. Allele frequency can vary drastically between genetic ancestries; therefore, it is recommended to account for it when using control populations to limit population stratification bias. Only 4 of 9 studies [11–13, 17] using control individuals who were different from family members matched case patients and controls with respect to genetic ancestry.

#### Assessment of Confidence in Candidate Genes or Variants

Sequencing artifacts can be mistaken for genetic variations. Prior to any variant identification, quality controls must be applied. All included studies described coverage control to validate appropriate coverage of regions of interest, and only 1 study did not describe genotyping quality control methods [20]. Bias can also result from sequencing individuals separately in different batches, as seemed to be the case for 6 studies [12–17]. Scott et al [13] accounted for this in their statistical analysis and adjusted it to limit its impact on the results. Finally, different sequencing techniques or libraries in case and control populations can produce detection bias [12, 13, 18–20].

Many tools can predict variant pathogenicity. Nine studies [11–13, 15–20] used a combination of multiple prediction tools and methods to select variants with the most putative effect on disease development. In terms of study design, 8 studies [10, 14–20] focused on or started with the analysis of genes known to be implicated in immune disorders, and all of them verified the biological credibility of candidate genes in previously published studies.

#### Statistics

Statistical methods were applied in 9 studies [10–14, 16–18, 21], and 4 of them corrected their results for multiple testing [12, 13, 16, 17]. Three studies described the proportion of the case population presenting variants in IEI genes [10, 14, 16], but the proportion of controls with mutated IEI genes was not calculated or not reported.

#### Results Validation

Results obtained in a discovery analysis were validated in an independent replication study in 4 articles [11, 13, 15, 21], and Salas et al [12] performed a pseudo replication using a different control population. To validate the causality of a genetic variation and its impact on protein function and disease development, functional analyses are necessary. It was performed in 7 of 12 studies [10, 11, 13, 15, 17, 20, 21].

#### Risk of Bias Assessment

Some biases are inherent to genetic studies and are common to all selected articles. All authors assumed full penetrance of the identified variants, a common hypothesis to identify novel causal genetic variation, but variable penetrance should be considered when the definite pathogenic role of each variant is assumed [23].

A risk of reporting bias was detected in 6 studies [10, 15, 18–21] in which it seemed authors might not have reported all results, positive and negative, obtained during the research project. Finally, risk of selection bias was detected in 4 studies with specific restrained patient selection, possibly limiting the generalization of the results to other, less restricted populations [13, 15, 18, 21].

#### Methodology Assessment Scoring Using Q-Genie Tool

We applied the Q-Genie scoring tool [24] to assess the methodology of all studies except case reports. All studies were evaluated as “good quality” according to this score (Table 3).

### Results Description

Individual results for each study are described in Table 2.

#### Diagnostic Yield of WES

The primary outcome for 2 studies [14, 16] was the diagnostic yield of WES in these patients. Overall, 20% and 61% of the described patients had variants in IEI genes, respectively. Notably, they did not restrict the analysis to the same exact gene panels. Borghesi et al [14] focused on 240 primary immunodeficiency (PID) genes associated with bacterial disease, whereas Kernan et al [16] used a broader gene panel of 430 IEI genes. Of the patients recruited by Kernan et al, 158 (48%) were previously healthy, as were the 176 patients studied by Borghesi et al [14]. Among those patients, 89 of 158 (56%) and 35 of 176 (20%) had variants in IEI genes. Taken together, 124 of the 334 previously healthy patients with sepsis (37%) had variants in IEI genes. Discrepancies between the results of these 2 studies can arise from different methodological approaches. As stated above, these authors did not restrict their analyses to the same gene panels. As the exact list of 240 PID genes associated with bacterial infection used by Borghesi et al [14] was not explicitly described, we could not compare the results with variants identified in these exact same genes in the population of Kernan et al [16]. Moreover, putatively pathogenic variants were not selected using the same pathogenicity annotation and classification; thus, comparisons between the 2 are is limited.

Three case reports [18–20] applied careful variant prioritization processes, and WES allowed the identification of variations in IEI genes that could explain the clinical phenotypes for all 3 cases. Finally, the primary objectives of Asgari et al [10] and Bendapudi et al [17] were not to evaluate the diagnostic yield of WES but to identify genetic associations with *P. aeruginosa* and purpura fulminans, respectively. Nevertheless, their analyses identified putatively disease-causing variants in 11 of 11 and 18 of 24 cases, respectively. These numbers were extracted from raw results of these 2 studies.

Overall, 7 studies focused on either all known IEI genes or specific subgroups of IEI genes. In these, 156 of 372 previously healthy patients (42%) had putatively disease-causing variants in IEI genes. These numbers were pooled together from studies applying different variant prioritization strategies, on patients with different infectious phenotypes. Only 2 of the studies were specifically designed to evaluate the diagnostic yield of WES. They should then be interpreted carefully.

#### Identification of Genetic Association With SBI

Two studies investigated genetic susceptibility to severe *P. aeruginosa* infection. One was a case report [20], and the other included 11 cases [10]. Their results did not outline the same genes (Table 2). Asgari et al [10] associated 12 genes bearing putatively pathogenic variants with *P. aeruginosa* infection (*EPHB6, DNMT3B, SPTBN4, SOX1, BTK, DCAF8L1, SHROOM2, HUWEI, ACRC, PJA1, NXF5,* and *AMMECR1*) and, when focusing on PID genes, outlined the enrichment of the complement pathway. In their case report, Zhang et al [20] identified a new compound heterozygous variant in the *IRAK4* gene responsible for the severity of the infection presented by their patient. It seems that severe *P. aeruginosa* infection justifies genetic investigations, as genetic defects were found in 7 of 12 patients (58%).

Two studies described genetic susceptibility to pneumococcal pneumonia [12, 21], combining 14 patients (n = 8 and n = 6, respectively). They did not identify the same genes associated with this disease. Salas et al [12] found 5 genes to be associated with empyema (*MEIS1, TSPAN15, OR9G9, MUC3A, MUC6,* and *APOB*), and Walker et al [21] identified *COQ6* as a new gene possibly associated with pneumococcal acute lower respiratory tract infections. The populations differed greatly between these 2 studies. Salas et al [12] selected 8 children with pneumococcal empyema in a Spanish population, whereas Walker et al [21] focused on children with pneumococcal acute lower respiratory tract infections in Papua New Guinea; the genetic ancestries of these 2 populations differ greatly, and their genetic susceptibility to disease could thus also be different.

Four studies [11, 14, 16, 19], including 1 case report [19], investigated patients who presented with severe sepsis due to multiple bacteria. Together, they accounted for 544 patients with sepsis and WES analysis; 372 were previously healthy. Three of the the studies [14, 16, 19] identified variations in known IEI genes. Taudien et al [11] underlined sepsis association with 5 major biological pathways, namely the Gαq, Toll, detection of stimulus, CDC42, and Her2 pathways.

The 4 other articles identified in this review studied various infectious phenotypes (*S. aureus* bacteremia [13], invasive meningococcal disease [15], purpura fulminans [17], and recurrent typhoid fever [18]). Because of these different infection types, results cannot be compared or aggregated.

Overall, the complement system was the biological pathway most frequently associated with SBI. It was highlighted in 3 studies [10, 16, 17] representing a total of 378 individuals with *P. aeruginosa* infection, sepsis, or purpura fulminans.

## DISCUSSION

We reviewed studies using a WES approach in previously healthy patients with SBI to identify genetic factors influencing the development of these life-threatening diseases. Most previous studies on this subject chose a narrow candidate gene approach [6, 7]. The use of NGS technologies such as WES allows the identification of new genetic defects without restricting the analysis to a few selected genes. We limited this review to studies focusing on previously healthy patients, as these patients should have a different genetic predisposition to SBI than patients with known immune deficiency or other dysimmune manifestations.

We identified 12 studies, including 3 case reports. They focused on specific well-defined phenotypes in homogenous cases. They studied different kinds of infections that could have different genetic predispositions, explaining the different results. Statistics were not always available and rarely corrected for multiple testing. Owing to the small sample size, statistical power was limited. A key point is the heterogeneity of the different studies included; indeed, 2 studies explored patients with pneumococcal disease, 2 focused on *P. aeruginosa* infections, 4 focused on sepsis due to multiple bacteria, and the remaining 4 each investigated SBI from different bacteria and with different phenotypes. In addition to various clinical phenotypes studied, many different analysis methods were applied. This diversity might explain why, apart from complement pathway identified in 3 studies [10, 16, 17] and the *IRAK4* gene identified in 2 case reports [19, 20], results involved various genetic defects in genes implicated in immune functions.

Among these various genetic defects identified through WES, some of them were in genes not yet linked to IEI (eg, *COQ6* [21], *GLS2* [13], and *SPLUNC1* [15]) These newly identified genes could bring knowledge in the pathophysiology of SBI. They illustrate the importance of NGS techniques to unravel unknown biological mechanisms. Even when authors considered the phenotypic heterogeneity of their studied patients, a recurrent conclusion was the diagnostic performance of WES in patients with SBI. Five articles [10, 14, 16, 19, 20] emphasized the importance of considering WES in clinical practice for patients with severe and/or recurrent bacterial infections to increase the odds of providing a genetic diagnosis. In this review, 42% of previously healthy patients with SBI had putatively disease-causing variants in IEI genes. While this number should be interpreted carefully due to differences in studied phenotypes and methodological approaches, it underlines the diagnostic yield of WES in patients with SBI, in accordance with conclusions from the 2 studies specifically designed to evaluate WES performance in diagnosis [14, 16].

Identifying an association between a genetic defect and SBI is not sufficient to formally implicate it in the pathophysiology of the disease. Indeed, as illustrated by Asgari et al [10], functional analyses are necessary for strict validation of potentially causal mutations. In this study, 1 patient presented a potentially disease-causing variant in the *C9* gene, known to be associated with meningitis, but had normal complement lytic activity in functional testing. Even a variant as plausible as this one is not necessarily the cause of the disease. Moreover, comparing results with those in control populations or healthy family members is also essential to validate results. Finally, to further validate the results, replication is needed with a new case population. This was rarely achieved in the studies presented in this review. This advocates for better collaboration between research teams working in similar fields to facilitate replication studies and increase sample size.

These studies chose different tools to prioritize variants, providing new insights for proper analysis of WES data. Variant effect prediction is complex, and multiple tools are available [25]. Guidelines were published in 2015 by the American College of Medical Genetics (ACMG) and the Association for Molecular Pathology (AMP) to classify variants according to their predicted pathogenicity [26]. These guidelines have not yet been applied to many studies but should be more integrated into WES analyses in the future. Other tools, such as burden test or tests derived from burden test (eg, the rare variant trend test [RVTT] [17]) were useful in some studies to group variants at the gene or pathway level and thus identify potentially implicated genes or pathways without significant results at the variant scale.

There are several limitations to this review. We restricted the selection to a specific type of study: WES in previously healthy patients who developed SBI. Many other studies have been published without restrictions to previously healthy patients but also included people with comorbid conditions or known immune deficiencies. It has recently become clear that immune deficiencies, allergies, and autoimmune disorders are intricate phenomena that could have a common genetic origin [4]. We did not include these dysimmune phenotypes in our review, considering previously healthy patients with SBI to be a very specific type of patient and probably a specific type of SBI predisposition representing a separate entity, with dysimmune disorders often carrying genetic variants in genes with IEI.

Overall, in this review, we describe well-conducted studies using NGS to identify genetic implications in the development of SBI. Data are still scarce, as they represent only 1186 patients worldwide (including 694 with WES data) with different infectious phenotypes. These studies highlight 2 interesting applications for WES: IEI genetic diagnosis in patients with SBI and identification of novel genetic association with genes not yet linked to infectious disease development or severity. Most previous works on genetic susceptibility to SBI chose a candidate gene approach. Further studies using these unrestricted NGS approaches would allow the identification of new genes involved in SBI, to better understand the biological mechanisms at play in severe infectious diseases. However, for the new genetic associations to be robust, these studies need to apply strict methodology and validation processes to large cohorts. To that end, collaboration between research teams investigating these rare diseases will be essential.

Taken together, studies reported here support the use of WES to identify new genes implicated in SBI development and to diagnose underlying genetic predisposition for these patients. As stated by previous studies, patients with SBI should be considered for genetic investigations using NGS [6]. NGS techniques are already used in clinical facilities to ease genetic diagnosis [27]. A better understanding of the mechanisms implicated in the development and severity of SBI is essential to identify novel therapeutic targets but also to improve prevention and early detection of these diseases, thus improving their prognosis.

## Supplementary Material

jiag006_Supplementary_Data

## Supplementary Data

Supplementary materials are available at *The Journal of Infectious Diseases* online (http://jid.oxfordjournals.org/). Supplementary materials consist of data provided by the author that are published to benefit the reader. The posted materials are not copyedited. The contents of all supplementary data are the sole responsibility of the authors. Questions or messages regarding errors should be addressed to the author.
