# Supplementary material for: Whole-Exome Sequencing for the Identification of Genetic Factors Implicated in Severe Bacterial Infections: A Systematic Review
Source: J Infect Dis. 2026 Jan 7;233(3):e600–10. doi: 10.1093/infdis/jiag006 (PMC13017026; doi:10.1093/infdis/jiag006)
Supplement: jiag006_Supplementary_Data [file jiag006_supplementary_data.docx]

# Supplementary material

## Supplementary Methods

We searched PubMed and Google Scholar for relevant articles published from January 2013 to January 2025. These sources were last searched on January 29^th^, 2025. We elaborated search strategies using keywords that seemed relevant to authors, resulting in an acceptable number of occurrences (<2000 records). The following search boxes were used:

- In PubMed: ("exome sequencing" OR "WES") AND (("infection") OR ("sepsis")) NOT "virus" NOT "mycobacterial" NOT "fungus"
- In PubMed: ("whole exome sequencing"[MeSH Terms] OR "whole exome sequencing" OR "WES") AND ("bacterial infections"[MeSH Terms] OR "severe bacterial infection" OR "sepsis" OR "septic shock") NOT fungus NOT virus NOT cancer NOT covid
- In Google Scholar: ("whole exome sequencing" OR "WES") AND ("bacterial infection" OR "bacterial infections") AND ("previously healthy" OR "otherwise healthy") AND (patients OR individuals) AND (diagnosis OR outcomes OR susceptibility)
- In Pubmed: rare AND variants AND exome AND bacterial infection.

We included studies that used WES to explore the genetic susceptibility of previously healthy individuals to bacterial infections. We included only studies that were accessible online and written in English.

We excluded studies with a candidate gene approach, such as targeted sequencing, gene panel analysis, or clinical exome sequencing. We focused only on bacterial infections, thus excluding mycobacterial, fungal, and viral infections. We also excluded articles that studied patients with a genetic diagnosis, a known susceptibility to bacterial infections, any other dysimmune manifestations or any other important clinical manifestations to focus only on articles studying SBI in previously healthy patients without a known predisposition for infections. We ultimately excluded commentaries and preprints.

One author (MG) screened the titles of all the retrieved records and then reviewed the abstracts of the eligible articles. After careful whole-text reading from the same author, relevant articles were finally selected. Data were collected from 1 author (MG).

We screened articles to collect methodological and result data. Sought outcomes were variants, genes, and/or pathways associated with SBI susceptibility and/or severity or the proportion of cases presenting with variants in genes known to be implicated in IEI or primary immune deficiencies (PID). For each article, we collected the name of the first author, year of publication, methods used for WES and quality control, patient phenotype and number of cases, control population description and number of controls, inclusion design, annotation tools used to predict variant pathogenicity, whether rare variants were filtered, use of gene panels and number of genes in these panels, validation techniques (Sanger sequencing and/or functional analyses), performance of familial analysis, replication studies, principal component analyses (PCA) to study patients’ genetic ancestries, statistical tests applied and use of correction for multiple testing in statistical analyses.

Studies were evaluated for bias by one author (MG) by carefully screening the articles. Risk of bias was evaluated following guideline suggestions for the evaluation of genetic studies.[1,2] Methodological quality evaluation was further performed by one author (MG) for each study using the Q-Genie scoring tool.[3] This tool was specifically designed to evaluate quality of genetic association studies included un systematic reviews. Score > 45 or > 40 indicate good quality studies with or without control groups, respectively. Studies were screened for selection bias due to case selection criteria, detection bias due to methods used to detect variants, publication and reporting bias, batch effect, bias due to sequencing techniques and coverage, population stratification, and bias arising from case/control definition.

A certainty assessment of the results was performed by screening articles for items listed in the guidelines emitted by MacArthur et al. in 2014[2] to investigate the causality of sequence variants in human disease. As stated, not every sequence variant is responsible for disease development in humans, and specific recommendations need to be followed to avoid wrongly linking genetic defects to diseases.

Table S1. PRISMA 2020 checklist for reporting systematic reviews

| **Section and Topic** | **Item #** | **Checklist item** | **Location where item is reported** |
| --- | --- | --- | --- |
| **TITLE** | | |  |
| Title | 1 | Identify the report as a systematic review. | P1 |
| **ABSTRACT** | | |  |
| Abstract | 2 | See the PRISMA 2020 for Abstracts checklist. | P3 |
| **INTRODUCTION** | | |  |
| Rationale | 3 | Describe the rationale for the review in the context of existing knowledge. | P5 |
| Objectives | 4 | Provide an explicit statement of the objective(s) or question(s) the review addresses. | P6 |
| **METHODS** | | |  |
| Eligibility criteria | 5 | Specify the inclusion and exclusion criteria for the review and how studies were grouped for the syntheses. | Supplementary P1-3 |
| Information sources | 6 | Specify all databases, registers, websites, organisations, reference lists and other sources searched or consulted to identify studies. Specify the date when each source was last searched or consulted. | Supplementary P1-3 |
| Search strategy | 7 | Present the full search strategies for all databases, registers and websites, including any filters and limits used. | Supplementary P1-3 |
| Selection process | 8 | Specify the methods used to decide whether a study met the inclusion criteria of the review, including how many reviewers screened each record and each report retrieved, whether they worked independently, and if applicable, details of automation tools used in the process. | Supplementary P2 |
| Data collection process | 9 | Specify the methods used to collect data from reports, including how many reviewers collected data from each report, whether they worked independently, any processes for obtaining or confirming data from study investigators, and if applicable, details of automation tools used in the process. | Supplementary P2 |
| Data items | 10a | List and define all outcomes for which data were sought. Specify whether all results that were compatible with each outcome domain in each study were sought (e.g. for all measures, time points, analyses), and if not, the methods used to decide which results to collect. | Supplementary P2 |
|  | 10b | List and define all other variables for which data were sought (e.g. participant and intervention characteristics, funding sources). Describe any assumptions made about any missing or unclear information. | Supplementary P2 |
| Study risk of bias assessment | 11 | Specify the methods used to assess risk of bias in the included studies, including details of the tool(s) used, how many reviewers assessed each study and whether they worked independently, and if applicable, details of automation tools used in the process. | Supplementary P2-3 |
| Effect measures | 12 | Specify for each outcome the effect measure(s) (e.g. risk ratio, mean difference) used in the synthesis or presentation of results. | P7 |
| Synthesis methods | 13a | Describe the processes used to decide which studies were eligible for each synthesis (e.g. tabulating the study intervention characteristics and comparing against the planned groups for each synthesis (item #5)). | P7 |
|  | 13b | Describe any methods required to prepare the data for presentation or synthesis, such as handling of missing summary statistics, or data conversions. | NA |
|  | 13c | Describe any methods used to tabulate or visually display results of individual studies and syntheses. | P7 |
|  | 13d | Describe any methods used to synthesize results and provide a rationale for the choice(s). If meta-analysis was performed, describe the model(s), method(s) to identify the presence and extent of statistical heterogeneity, and software package(s) used. | P7 |
|  | 13e | Describe any methods used to explore possible causes of heterogeneity among study results (e.g. subgroup analysis, meta-regression). | P7 |
|  | 13f | Describe any sensitivity analyses conducted to assess robustness of the synthesized results. | NA |
| Reporting bias assessment | 14 | Describe any methods used to assess risk of bias due to missing results in a synthesis (arising from reporting biases). | NA |
| Certainty assessment | 15 | Describe any methods used to assess certainty (or confidence) in the body of evidence for an outcome. | Supplementary P2-3 |
| **RESULTS** | | |  |
| Study selection | 16a | Describe the results of the search and selection process, from the number of records identified in the search to the number of studies included in the review, ideally using a flow diagram. | P8 and figure S1 |
|  | 16b | Cite studies that might appear to meet the inclusion criteria, but which were excluded, and explain why they were excluded. | NA |
| Study characteristics | 17 | Cite each included study and present its characteristics. | Table 1 |
| Risk of bias in studies | 18 | Present assessments of risk of bias for each included study. | P11 and table 3 |
| Results of individual studies | 19 | For all outcomes, present, for each study: (a) summary statistics for each group (where appropriate) and (b) an effect estimate and its precision (e.g. confidence/credible interval), ideally using structured tables or plots. | Table 2 |
| Results of syntheses | 20a | For each synthesis, briefly summarise the characteristics and risk of bias among contributing studies. | Table 3 |
|  | 20b | Present results of all statistical syntheses conducted. If meta-analysis was done, present for each the summary estimate and its precision (e.g. confidence/credible interval) and measures of statistical heterogeneity. If comparing groups, describe the direction of the effect. | P12-14 |
|  | 20c | Present results of all investigations of possible causes of heterogeneity among study results. | P12-14 |
|  | 20d | Present results of all sensitivity analyses conducted to assess the robustness of the synthesized results. | NA |
| Reporting biases | 21 | Present assessments of risk of bias due to missing results (arising from reporting biases) for each synthesis assessed. | NA |
| Certainty of evidence | 22 | Present assessments of certainty (or confidence) in the body of evidence for each outcome assessed. | P10-11 and table 3 |
| **DISCUSSION** | | |  |
| Discussion | 23a | Provide a general interpretation of the results in the context of other evidence. | P14-16 |
|  | 23b | Discuss any limitations of the evidence included in the review. | P16-17 |
|  | 23c | Discuss any limitations of the review processes used. | P16-17 |
|  | 23d | Discuss implications of the results for practice, policy, and future research. | P17 |
| **OTHER INFORMATION** | | |  |
| Registration and protocol | 24a | Provide registration information for the review, including register name and registration number, or state that the review was not registered. | NA |
|  | 24b | Indicate where the review protocol can be accessed, or state that a protocol was not prepared. | NA |
|  | 24c | Describe and explain any amendments to information provided at registration or in the protocol. | NA |
| Support | 25 | Describe sources of financial or non-financial support for the review, and the role of the funders or sponsors in the review. | P20 |
| Competing interests | 26 | Declare any competing interests of review authors. | P20 |
| Availability of data, code and other materials | 27 | Report which of the following are publicly available and where they can be found: template data collection forms; data extracted from included studies; data used for all analyses; analytic code; any other materials used in the review. | P17 |

*From:*  Page MJ, McKenzie JE, Bossuyt PM, Boutron I, Hoffmann TC, Mulrow CD, et al. The PRISMA 2020 statement: an updated guideline for reporting systematic reviews. BMJ 2021;372:n71. doi: 10.1136/bmj.n71

## Variant prioritization

To prioritize variants, 10 studies out of 12 focused on rare variants (n=9)[4–12] with different minor allele frequency (MAF) cut-offs (Table 2), or variants absent in public databases (n=1).[13] One study explored both rare and common variants in different steps of the analysis.[14] All studies focused on predicted deleterious variants for protein function. Nine studies[4,5,8,9,11–15] used different tools to annotate variants according to their predicted pathogenicity and impact on disease development in humans (Table 2). The most commonly used tools were CADD[16] (n=6), SIFT[17] (n=5), PolyPhen-2[18] (n=3), and ACMG AMP[19] criteria (n=3). Other tools used are indicated in Table 2.

Five studies focused on gene panels[4,6–8,11] compatible with disease phenotypes, comprising 25 to 430 genes previously known to be associated with bacterial infection development in humans.

Finally, five studies further prioritized genes to focus on those most likely to be involved in SBI development. They used gene set enrichment analyses (GSEA, n = 3)[6,12,14] to select genes impacting pathways compatible with the clinical phenotype and burden test (n = 3)[11,14,15] to identify genes (n=2)[14,15] or gene sets (n=1)[11] more frequently bearing variants in cases or in controls.

## Supplementary references

1. Little J, Higgins JPT, Ioannidis JPA, et al. Strengthening the reporting of genetic association studies (STREGA): an extension of the STROBE Statement. Hum Genet. **2009**; 125(2):131–151.

2. MacArthur DG, Manolio TA, Dimmock DP, et al. Guidelines for investigating causality of sequence variants in human disease. Nature. **2014**; 508(7497):469–476.

3. Sohani ZN, Meyre D, De Souza RJ, et al. Assessing the quality of published genetic association studies in meta-analyses: the quality of genetic studies (Q-Genie) tool. BMC Genet. **2015**; 16(1):50.

4. Hassan FU, Aljeldah MM, Fozia F, et al. Whole Exome Sequence Analysis for Inborn Errors of IL-12/IFN-γ Axis in Patient with Recurrent Typhoid Fever. Kandeel M, editor. BioMed Res Int. **2023**; 2023:1–6.

5. Altammar F, Alshamali M, Alqunaee M, Alali AJ, Elshafie RM, Al-Herz W. A case report of a patient with recurrent and severe infections highlighting the importance of considering inborn errors of immunity. Front Pediatr. **2024**; 12:1340367.

6. Asgari S, McLaren PJ, Peake J, et al. Exome Sequencing Reveals Primary Immunodeficiencies in Children with Community-Acquired Pseudomonas aeruginosa Sepsis. Front Immunol [Internet]. **2016** [cited 2021 Jan 7]; 7. Available from: http://journal.frontiersin.org/Article/10.3389/fimmu.2016.00357/abstract

7. Borghesi A, Trück J, Asgari S, et al. Whole-exome sequencing for the identification of rare variants in primary immunodeficiency genes in children with sepsis - a prospective population-based cohort study. Clin Infect Dis Off Publ Infect Dis Soc Am. **2020**; .

8. Kernan KF, Ghaloul-Gonzalez L, Vockley J, et al. Prevalence of Pathogenic and Potentially Pathogenic Inborn Error of Immunity Associated Variants in Children with Severe Sepsis. J Clin Immunol. **2022**; 42(2):350–364.

9. Mashbat B, Bellos E, Hodeib S, et al. A Rare Mutation in SPLUNC1 Affects Bacterial Adherence and Invasion in Meningococcal Disease. Clin Infect Dis Off Publ Infect Dis Soc Am. **2020**; 70(10):2045–2053.

10. Walker EC, Javati S, Todd EM, et al. Novel coenzyme Q6 genetic variant increases susceptibility to pneumococcal disease. Nat Immunol. Nature Publishing Group; **2024**; 25(12):2247–2258.

11. Bendapudi PK, Nazeen S, Ryu J, et al. Low-frequency inherited complement receptor variants are associated with purpura fulminans. Blood. **2023**; :blood.2023021231.

12. Taudien S, Lausser L, Giamarellos-Bourboulis EJ, et al. Genetic Factors of the Disease Course After Sepsis: Rare Deleterious Variants Are Predictive. EBioMedicine. **2016**; 12:227–238.

13. Zhang F, Wang Z, Men S, Zhang J, Wang L. Two novel compound heterozygous loss-of-function mutations cause fetal IRAK-4 deficiency presenting with Pseudomonas Aeruginosa sepsis. Clin Immunol. **2024**; 265:110268.

14. Salas A, Pardo-Seco J, Barral-Arca R, et al. Whole Exome Sequencing Identifies New Host Genomic Susceptibility Factors in Empyema Caused by Streptococcus pneumoniae in Children: A Pilot Study. Genes [Internet]. **2018** [cited 2021 Jan 14]; 9(5). Available from: https://www.ncbi.nlm.nih.gov/pmc/articles/PMC5977180/

15. Scott WK, Medie FM, Ruffin F, et al. Human genetic variation in GLS2 is associated with development of complicated Staphylococcus aureus bacteremia. PLoS Genet. **2018**; 14(10):e1007667.

16. Rentzsch P, Witten D, Cooper GM, Shendure J, Kircher M. CADD: predicting the deleteriousness of variants throughout the human genome. Nucleic Acids Res. **2019**; 47(D1):D886–D894.

17. Kumar P, Henikoff S, Ng PC. Predicting the effects of coding non-synonymous variants on protein function using the SIFT algorithm. Nat Protoc. **2009**; 4(7):1073–1081.

18. Adzhubei I, Jordan DM, Sunyaev SR. Predicting Functional Effect of Human Missense Mutations Using PolyPhen-2. Curr Protoc Hum Genet Editor Board Jonathan Haines Al. **2013**; 0 7:Unit7.20.

19. Richards S, Aziz N, Bale S, et al. Standards and Guidelines for the Interpretation of Sequence Variants: A Joint Consensus Recommendation of the American College of Medical Genetics and Genomics and the Association for Molecular Pathology. Genet Med Off J Am Coll Med Genet. NIH Public Access; **2015**; 17(5):405.
